# Visual scanning patterns of a talking face when evaluating phonetic information in a native and non-native language

**DOI:** 10.1371/journal.pone.0304150

**Published:** 2024-05-28

**Authors:** Xizi Deng, Elise McClay, Erin Jastrzebski, Yue Wang, H. Henny Yeung

**Affiliations:** Department of Linguistics, Simon Fraser University, Burnaby BC, Canada; CNRS: Centre National de la Recherche Scientifique, FRANCE

## Abstract

When comprehending speech, listeners can use information encoded in visual cues from a face to enhance auditory speech comprehension. For example, prior work has shown that the mouth movements reflect articulatory features of speech segments and durational information, while pitch and speech amplitude are primarily cued by eyebrow and head movements. Little is known about how the visual perception of segmental and prosodic speech information is influenced by linguistic experience. Using eye-tracking, we studied how perceivers’ visual scanning of different regions on a talking face predicts accuracy in a task targeting both segmental versus prosodic information, and also asked how this was influenced by language familiarity. Twenty-four native English perceivers heard two audio sentences in either English or Mandarin (an unfamiliar, non-native language), which sometimes differed in segmental or prosodic information (or both). Perceivers then saw a silent video of a talking face, and judged whether that video matched either the first or second audio sentence (or whether both sentences were the same). First, increased looking to the mouth predicted correct responses only for non-native language trials. Second, the start of a successful search for speech information in the mouth area was significantly delayed in non-native versus native trials, but just when there were only prosodic differences in the auditory sentences, and not when there were segmental differences. Third, (in correct trials) the saccade amplitude in native language trials was significantly greater than in non-native trials, indicating more intensely focused fixations in the latter. Taken together, these results suggest that mouth-looking was generally more evident when processing a non-native versus native language in all analyses, but fascinatingly, when measuring perceivers’ latency to fixate the mouth, this language effect was largest in trials where only prosodic information was useful for the task.

## Introduction

When comprehending speech, listeners depend on more than just what they hear. Useful speech information is encoded in many sensory modalities besides audition, like visual cues from the interlocutor’s face, which can enhance auditory speech comprehension [1]. For example, when auditory speech fails to provide perceivers with sufficient acoustic information in noisy and degraded circumstances, visual cues from the face emerge as a complementary system for comprehending speech [2–5]. The use of visual speech cues can also be seen when auditory speech is not degraded, like when perceivers are presented with mismatched audio-visual presentation, and report hearing ‘fused’ audio-visual percepts [6]. In addition, even when listeners are presented with clear and audio-visually consistent speech, but this speech is cognitively challenging to process—because it is in an unfamiliar language, dialect or in a semantically and syntactically complex context in their native language—then visual speech can also assist with processing [7].

There are several reasons why visual cues exert enhancement of auditory speech comprehensibility, and a leading idea is that critical speech cues are embedded in particular areas in the face, like the mouth. For example, a number of studies have shown that attention to the mouth of a talking face emerges early in development, from early in infancy [8–11]. Likewise, research has found that adults also pay attention to the mouth during speech tasks, for example when identifying segments of CV syllables [12], or when identifying which face is the source of auditory speech [13]. These results suggest that, for both infants and adults, the mouth area of a talking face provides useful cues for decoding speech information. Indeed, previous research has well established that visible articulatory movements of the speaker’s mouth (e.g., lips), resulting in different vocal tract configurations for different segments, are useful sources for segmental perception [14–18].

While the mouth can be helpful for segmental speech perception, it may not provide direct visual cues for prosodic perception, presumably because the production of prosodic variations does not rely on vocal tract configurations. Instead, the other parts of the face (e.g., eyebrows, head) have been found to be helpful for emotion and social interaction [19–22], particularly for linguistic prosody [23–28]. For example, when auditory prosodic contrasts were articulated, there was an increase of eyebrow movements and head rotations in narrow focused statements and echoic questions in English [23]. Likewise, head and eyebrow movements were also found to be aligned with different pitch trajectories when producing the four lexical tones in Mandarin [25], as well as aligned with pitch and amplitude variation of a talker’s voice when producing Japanese sentences [27]. More importantly, listeners are able to utilize gestural cues in both eye and general face areas to extract relevant speech information at the prosodic level. For example, [27] found, when head movements were present, participants were able to identify more Japanese syllables than when head movement was not seen. In addition, [23] found that the upper part of a face has stronger cue value in the detection of word-level prosodic prominence in a three-word sentence. [26] further divided prosody into either intonation (“We won,” versus “We won?”) or sentential stress (i.e. contrastive focus on words in a sentence, e.g. “WE won,” versus “We WON”), and found that when participants were asked to make judgments specifically about intonation patterns by watching a silent talking face, their gaze duration at the upper part of the face (the forehead and the eyes) was longer than when making decisions about segments. In this paper, it also appeared that participants tended to look more at the upper than lower part (including the mouth and chin) of the face when detecting sentential stress patterns (although this effect was slight, and statistically non-significant), and additionally, there was a more evenly distributed looking pattern for sentential stress than when participants were looking for segmental information (i.e. cues differentiating different segments).

To put all these prior studies together, these results suggest that visual information about prosody cues, such as sentential stress and intonation, can be observed as perceivers fixate the general face area (more equal looking to the upper and lower part of the face) or the upper area (particularly for intonation), while information about segments can be extracted as perceivers fixate more the lower part of the face, including the mouth region. There are a number of other factors, however, that influence how perceivers extract information from a talking face. One additional factor is related to the talker, like whether the perceived face is talking in the perceiver’s native or non-native language. In several eye-tracking studies, there seems to be an overall greater proportion of fixations at the mouth in a non-native language compared to a native language [29], a pattern also seen for listeners who have high L2 proficiency [30]. Another important factor to consider is different types of fixation behaviors from the perceiver. While prior work has mostly examined perceivers’ proportion of time fixating different parts of the face (i.e., the mouth versus the eyes), there are several other potentially informative measures of gaze behaviour. For example, both classic [5] and more recent studies [31] have examined how perceivers tend to scan different parts a talking face—measuring the locations and amplitudes of saccades as perceivers scan salient features of a face like the eyes and mouth—and have shown that the dispersion of saccades (i.e., the average amplitude of a saccade as it scans a face) can change as a function of how clearly speech is heard [31]. Other studies have examined fixation latencies, or the time needed to launch a visual search to a relevant part of the face (i.e., the mouth), which can be calculated seeing when gaze shifts from the eyes (perhaps when a face is not talking), to the launching of the first saccade to the mouth once the face begins to talk. For example, this measure has been used in previous studies [32, 33] to suggest how strongly perceivers may expect upcoming visual information to be task-relevant.

### The current study

The present study investigated eye gaze when perceivers processed different types of phonetic information in both familiar (native) and unfamiliar (non-native) languages. More specifically, this study focused on how perceivers encode stored speech information in their memory based on their linguistic experience and where they expect to see on a speaker’s face in order to identify such speech properties. This study integrated across the previous studies reviewed above in several novel ways. First, unlike previous studies [27, 28], we included different types of phonetic information (segmental, prosodic) in a single experiment to directly compare the eye gaze patterns when processing segmental and prosodic cues which are expected to involve different facial regions. Second, we asked how visual scanning for these different types of phonetic information might also differ when perceiving a native versus a non-native language. Particularly, we were interested in whether there would be any interactions between language and phonetic information type, which have only been investigated separately in previous studies. Third, inspired by previous studies such as [5, 31–33], we examined not just where on a face that perceivers scanned, but also the speed of their search for linguistic information and overall scanning patterns (i.e., saccade amplitudes). Below we elaborate our study design, showing how we investigated how perceivers scan a talking face for different types of speech information.

The current design is related to [29], where monolingual English speakers were required to encode visual features in two videos and match the information in a subsequently presented audio clip with one of the previous videos. In our study, native English speakers were required to view a talking face while perceiving segmental and/or prosodic information. Specifically, they were presented with two auditory-only sentences that may have differed in words (i.e., segments), or in contrastive phrasal stress for particular words in the sentences (i.e., prosody), or in both words and contrastive stress. As such, our experiment participants needed to encode the auditory differences, and were then presented video-only talking faces. While searching for useful visual cues, their gaze and scanning patterns were recorded using an eye-tracker. In the end, participants were requested to identify which sentence (if they were in fact different) was uttered in the video. This was done in either English or Mandarin (a non-native, unfamiliar language to all participants). No instructions or hints were provided to the participants with regard to where they should look or how the sentences may differ. The rationale underlying this design is that we intended to examine perceivers’ expectation for searching different facial areas—that is, where they anticipated different linguistic information could potentially be embedded—after they encoded auditory differences.

This design also allowed us to explore visual scanning of a talking face while participants were actively searching for different types of linguistic information. We start by analyzing behavioural results from this task as an estimate of task difficulty, and then conducted three analyses of listeners’ looking patterns to understand how looking could predict task accuracy. First, we analyzed fixation proportions, or the proportion of dwell time spent fixating the eyes and mouth areas relative to the whole face. Consistent with prior work [26], we hypothesized greater mouth-looking when identifying segmental information, and comparatively less mouth-looking when participants were searching for prosodic information. When processing the native language (English), we again predicted overall greater looking to the mouth than the eyes, consistent with prior work [29], and further hypothesized an accentuation of this mouth-looking for the non-native stimuli (Mandarin).

Second, we analyzed saccade amplitude scanning patterns, or whether participants had more diffused scanning of the whole face versus more focused eye gaze at one particular area. In line with the predictions of fixation proportions, we predicted that there would be a more diffused scanning pattern when processing prosodic information, reflected by greater saccade amplitude, whereas there would be more focused looking when processing segmental information, suggested by smaller saccade amplitude. Likewise, we further supposed that more focused scanning pattern would be found when searching for non-native phonetic cues.

Third, we analyzed fixation latencies to move to the mouth as a measure of participants’ expectation that the mouth area contained critical information. In general, this fixation latency may reflect the initial identification of the auditory differences, and their salience in the memory. That is, successful detection of any auditory differences in the two sentences may cause listeners to more swiftly attend to the mouth and search for the matching visual cues in the portion of the trial with the silent visual search. Conversely, if participants were not sure about where the differences lay, or they mistakenly identified the two auditory sentences as the same, this may result in a delay of looking to the mouth. Our hypothesis thus was also that perceivers would make faster saccades to the mouth when looking for segmental, as opposed to prosodic information if segmental differences are easier for participants to identify in the mouth [26–28]. A corollary hypothesis for the language analysis would be that perceivers would have shorter fixation latencies in English compared to Mandarin trials, because they might more easily be able to link auditory memory traces to visual information in their native language, and move to the mouth.

In addition to separate main effects of phonetic information type (segmental versus prosodic cues) and language (native versus non-native), we also looked for interactions. In terms of our three analyses, we predicted that there might be a smaller difference between the segmental and prosodic conditions in Mandarin than in English. One reason for this prediction is that Mandarin prosody may be more difficult to identify than English prosody for our perceivers, given that participants had no lexical access when listening to Mandarin, and so perceivers may have defaulted to increased mouth-looking as a standard strategy in both segmental and prosodic conditions, obscuring the by-condition differences. Another possibility is that English listeners may process Mandarin stimuli in non-linguistic ways, given its novelty, and thus alter how segmental or prosodic cues guide attention to the eyes and other facial areas, making the difference between processing prosody and segments smaller in Mandarin. Any of these phenomena might result in an interaction between language and information type in those dimensions, such as more fixations at the mouth, smaller saccade amplitude and/or later fixation latency when processing prosodic information in Mandarin than that in English, compared to processing segmental information.

## Materials and methods

### Participants

The current study was approved by the Office of Research Ethics at our university. Twenty-four native English speakers (2 males; 22 females), aged from 19 to 23 (*mean* = 21.2, *SD* = 1.5), were recruited from our university between March 2019 and March 2020. The sample size is similar to the one in [29] where they had 30 participants for each language group. Written consent was obtained from these participants after presenting them with a hard copy of a consent form where certain information was included, such as how the experiment would be conducted, the potential benefits of participation and how their privacy would be protected, etc. For a preliminary intake form, potential participants filled out a questionnaire asking their age, place of birth (and age of arrival in Canada if relevant), as well as the age of acquisition and years spent learning foreign languages, a ranking of dominance in these languages, and a description of their musical experience. For this study, only native English speakers were tested, and anyone who had acquired Mandarin or other tonal languages (such as Cantonese) as either a native language or second language was not recruited for this study. All participants had normal or corrected-to-normal vision, and none had hearing impairments or language-related pathologies such as dyslexia. Participants received $10 or course credit for participation in this 1-hour study. The authors had access to the identifying information of the participants after the data collection, but only for the purpose of matching participants’ names and dates of the appointments in the booking system with other data obtained through questionnaires and the experiment. All the identifying information will only be stored in our lab for up to 7 years.

### Stimuli

**Fig 1:**
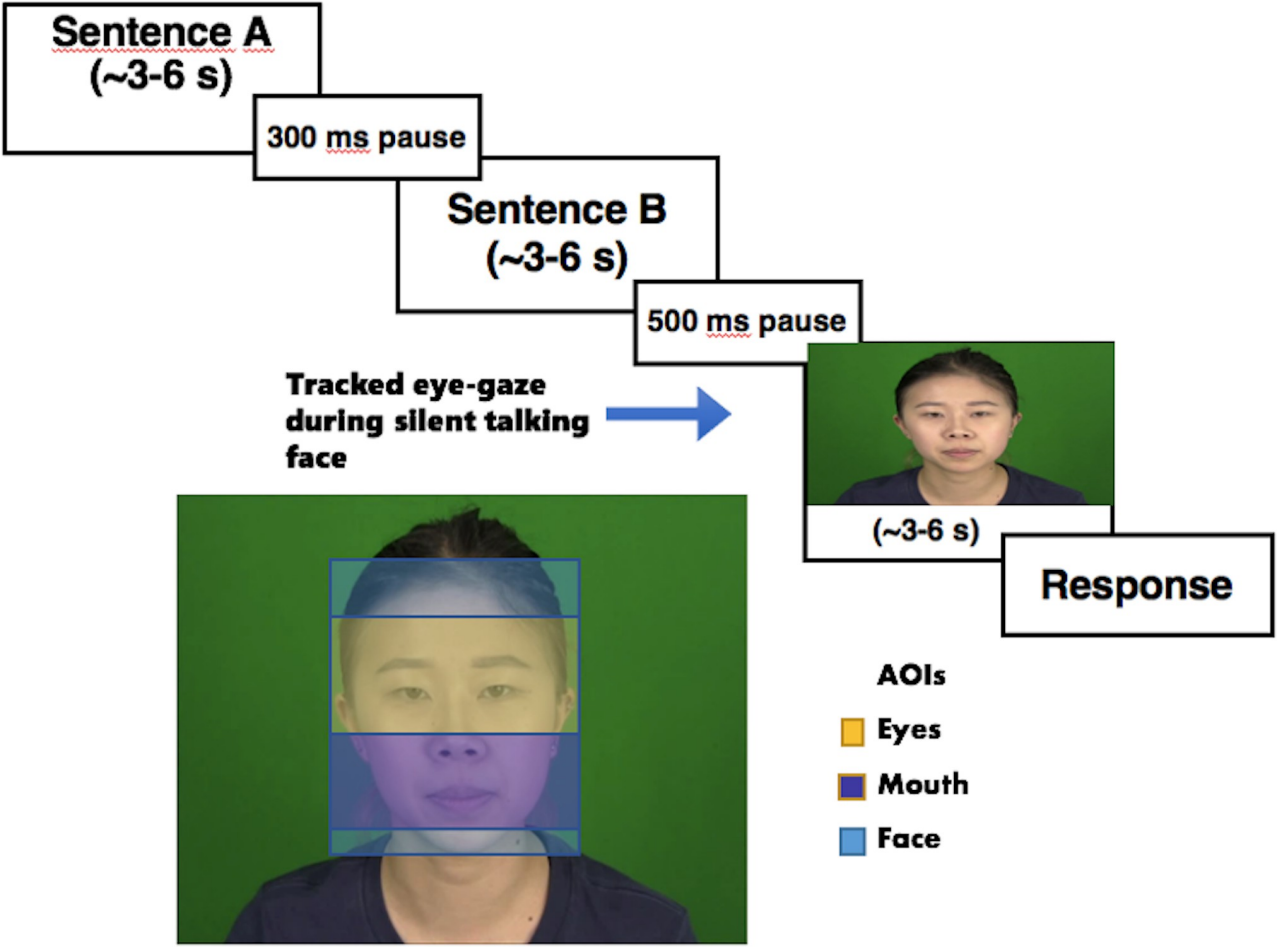
The structure of a trial. Participants first heard a sentence in either English or Mandarin followed by another sentence in the same language. Then, they saw a silent talking face and began scanning the face for useful speech cues in order to make a decision about which sentence matched the video. The trial ended when the response was recorded, or when the video ended (whichever was later).

Each trial consisted of two auditory sentences and one silent video, which showed a brief clip of a face articulating an auditory sentence. A female, simultaneous bilingual speaker of Canadian English and Taiwanese Mandarin recorded all auditory and visual stimuli, and these trials were arranged into one of four conditions (see Fig 1 and Table 1). The speaker in this study has given written informed consent (as outlined in PLOS consent form) to publish the details of her facial information in Fig 1 in this manuscript. In trials from the *Baseline* condition, both auditory sentences were the same and the silent video matched this sentence. In experimental trials, three conditions were run where auditory trials differed from each other, and the silent video only matched one of them: In the *Prosody* condition, the two auditory sentences differed in which words were stressed using contrastive focus, but both sentences had the same words; in the *Segment* condition, sentences differed in which words were used (having visually distinct consonants and vowels), but both sentences had the same pattern of sentence intonation; in the *Both* condition, sentences difference in both contrastive prosody and segments. In each trial, the two sentences were either both in English (the native language) or both in Mandarin Chinese (a non-native language).

**Table 1:** Stimuli from an example topic in English and in Mandarin.

Example Sentences	Condition
**Reference Sentence:** No, **JESS** found her **PLAIN** dress for **KYLA’S** wedding.	
No, **JESS** found her **PLAIN** dress for **KYLA’S** wedding.	Baseline
No, Jess **FOUND** her plain **DRESS** for Kyla’s **WEDDING**.	Prosody
No, **ANNE** found her **BLUE** dress for **JIMMY’S** wedding.	Segments
No, Anne **FOUND** her blue **DRESS** for Jimmy’s **WEDDING**.	Both
**Reference Sentence:** 不, 小**李**想给 他的大**姐**买 只 **红**色的 手表。	
不, 小**李**想给 他的大**姐**买 只 **红**色的 手表。 Bu4, xiao3 **LI3** xiang3 gei3 ta1 de da4 **JIE3** mai3 zhi1 **HONG2** se4 de shou3 biao3. “No, Li wants to buy his elder sister a red watch.”	Baseline
不, 小李**想**给他的大姐**买**只红色的**手**表。 Bu4, xiao3 Li3 **XIANG3** gei3 ta1 de da4 jie3 **MAI3** zhi1 hong2 se4 de **SHOU3** biao3. “No, Li wants to buy his elder sister a red watch.”	Prosody
不, 小**马**想给他的大**嫂**买只 **银**色的手表。 Bu4, xiao3 **MA3** xiang3 gei3 ta1 de da4 **SAO3** mai3 zhi1 **YIN2** se4 de shou3 biao3. “No, Ma wants to buy his sister in-law a silver watch.”	Segments
不, 小马**想**给他的大嫂**买**只银色的**手**表。 Bu4, xiao3 Ma3 **XIANG3** gei3 ta1 de da4 sao3 **MAI3** zhi1 yin2 se4 de **SHOU3** biao3. “No, Ma wants to buy his sister in-law a silver watch.”	Both

Note. Conditions in this example are defined relative to the reference sentence; if the reference was played first, then trial’s condition would be determined by the target sentence as the second one played. **Bold** (English) / underline (Mandarin) indicate words (or syllables) with contrastive stress intonation.

All stimuli were created in quadruplet groups, hereafter referred to as “topics.” An example of English and Mandarin stimuli from one topic is presented in Table 1, and the S1 Appendix in Supporting Information lists the full sets of stimuli used in both English and Mandarin. There were 4 sentences in each topic and 6 topics for each language, resulting in 48 sentences in total (4 sentences * 6 topics * 2 languages). Stimuli were controlled as closely as possible given the very different syntactic and lexical properties across languages: The length of the English sentences ranges from 11 to 18 syllables, with a mean of 14 syllables, while that of the Mandarin sentences ranges 14 from to 16 syllables, with a mean of 15 syllables. In the *Segment* condition, the first critical word in English stimuli that differentiates two sentences was after 1 to 6 syllables the from the beginning of the sentences, with a mean of 2.3 syllables. The corresponding word in Mandarin stimuli was after 1 to 5 syllables from the beginning, with a mean of 3 syllables. In the *Prosody* condition, the first critical word in English stimuli that differentiates two sentences was after 2 to 6 syllables the from the beginning of the sentences, with a mean of 3.5 syllables, while for Mandarin stimuli this was after 1 to 6 syllables from the beginning, with a mean of 3.2 syllables. There were three critical words in each sentence. The reason for having multiple critical words was to make this task less challenging, particularly in the non-familiar language condition, ensuring that target cues would not be missed.

Stimuli were recorded using a high-definition camcorder (Canon Vixia HF30) at 29 fps. Concurrent high-quality audio was recorded using a Shure KSM109 microphone at 48 kHz in a sound-attenuated booth. Stimuli were recorded with a preceding context sentence that established a discourse (e.g., “Did Anne find her blue dress for Jimmy’s wedding?”), and then the target sentence with contrastive focus was recorded (e.g., “No, **JESS** found her **PLAIN** dress for **KYLA’S** wedding”). All sentences were presented to the talker via computer slides.

The speaker started with a closed mouth and a neutral eyebrow position, and was further instructed to speak naturally with neutral facial expressions and minimal head movements. Final English stimuli were selected by a native English speaker, while Mandarin stimuli were selected by a native Mandarin speaker to maintain similar sentence durations across a topic, as well as to maintain overall quality of auditory and visual canonicity according to the condition description (e.g., was contrastive focus used correctly in the sound of her voice and in her face, as subjectively perceived by the stimuli selectors). Final versions of each English sentence were around 3–4 seconds in length, while Mandarin sentences were around 5–6 seconds. Each video had a resolution of 1024 * 768 pixels^2^ (24.1 * 23.5 squared degrees of visual angle).

### Procedure

After completing the preliminary demographic questionnaire, participants gave consent to participate in a 1-hour study. First, each participant, seated in front of a 17-in monitor with an eye-to-screen distance of 70 cm, was calibrated in an Eyelink 1000 eye-tracker in binocular mode (SR Research) using a standard nine-point procedure on a 1280 * 1024 pixels^2^ screen. Afterwards, instructions were given both on the screen and verbally by the experimenter, which informed participants that they would be presented with two auditory sentences followed by a single silent video that matched one (or both) of the audio-only stimuli in each trial. As shown in Fig 1, each trial began with presentation of both auditory sentences (with a 300 ms ISI), which were both in the same language. During this, the participants were instructed to look at an orange square on the screen while they were listening to the audio sentences. The square was situated in the eye area of the talking face that appeared later in the trial. This was followed by a 500 ms pause, and then the square turned blue before a video of a silent talking face was played. Participants’ eye gaze thus tended to start with the same pattern, beginning in the eye area, and then shifting to the mouth. This design is related to our analysis of fixation latency, since our study assesses their expectations about looking for visual cues to retrieve the pre-encoded auditory differences and pre-cuing them to a fixation point would facilitate our measurement of how strongly they associated those auditory differences by searching at various parts of the face, like the mouth (which is the main area they focused on). If they were extracting cues from the eyebrows after the start of the video, it could still be reflected by a delayed shift to the mouth. If they expected the lips to be useful, it could be reflected by a prompter shift to the mouth. During video playback participants could select a keyboard button to indicate which of the audio recordings (or both) matched the video they saw, in which case, the video continued to play to the end, and then advanced automatically to the next trial. If no responses were made during the video, they were instructed to make a choice when the video ended, which then triggered the beginning of the next trial. Participants could thus respond anytime from the beginning to the end of stimulus presentation. The three AOIs were depicted in Fig 1 and are based on the facial motion reflectors in [34]. Our eye area, occupying an area of 10.4 * 6.4 deg^2^, included the eyes and eye-brows (their Area 1), the mouth (10.4 * 5.5 deg^2^) only included the lips and the surrounding area (their Area 3), and our general face area (10.4 * 14.9 deg^2^) included the chin (their Area 4). What we focused on was the contribution of eye and mouth areas in particular to decoding segmental and prosodic information following [29] (although our mouth and eye areas are a little broader than theirs), rather than being concerned with broader upper and lower parts of the face like in [26, 28] (their lower part of the face also included the chin area).

For each participant, there were seven practice trials, which had trials where the video matched the first or second sentence, and where both sentences were identical. Every participant was instructed before the practice trials that there were segmental or prosodic differences in the two auditory sentences (or sometimes that the two sentences were identical) and thus they should press the left or right arrow button to indicate which video matched the silent video (i.e., the first or second sentence, respectively), or press the down arrow button to indicate that they were the same sentence. After the practice trials, the experiment began. Recall that each topic (see Table 1) contained four sentences, and so there were 16 possible pairings of sentences per topic in our trial design (e.g., S1-S1; S1-S2; S2-S1, etc.). Each participant heard each sentence seven times (e.g. S1 appeared in the following pairings: S1-S1, S1-S2, S2-S1, S1-S3, S3-S1, S1-S4, S4-S1). There were 4 blocks of 24 trials with untimed breaks in-between: Two consecutive blocks were in English, and two in Mandarin (counterbalanced across participants).

With six topics in each language, there were a total of 192 possible trials (16 pairings *6 topics *2 languages). However, each participant only received 96 trials in order to keep the experiment to a reasonable length. Across all participants, all unique 192 pairings were presented. Trials within a single participant were chosen such that both possible orders for a pair of auditory sentences (e.g., S1-S2 and S2-S1) were presented, once where the visual face matched one auditory sentence (i.e., once matching S1 and once matching S2). This ensured that data from both visual faces was collected within a single participant. Additionally, the order of trials was such that two trials from the same topic could not be within 3 intervening trials (and not within 4 or 6 intervening trials if one or both of the auditory sentences, respectively, was repeated). To achieve this, two pseudorandomized lists of 48 trials were generated (List 1 and List 2), each evenly distributed over the topics (8 trials from each topic), the number of trials in each condition (16 per language), and the number of visual matches to either the first or second sentence for non-Baseline trials (72 non-baseline trials for each participant and 36 for each language; 18 per language matching the first auditory sentence; 18 per language matching the second auditory sentence). Two more lists (List 1x and List 2x) contained trials in a reverse order from those two original lists. Across 24 participants, half were presented trials in the order determined by List 1 and 2 (*n* = 6 in English and Mandarin, respectively; *n* = 6 in Mandarin and English, respectively), while half were presented trials in the orders from Lists 1x and 2x (again, counterbalancing for language). The order of the languages was also counterbalanced across experiment halves: For half of the participants, the first two experimental blocks were in English, while the last two blocks were in Mandarin, with the reverse order for the other participants.

## Results

Following one of our reviewers’ feedback, the results from the *Baseline* condition will not be included in this paper. The results were analyzed in four parts: (1) behavioural responses, using response time and accuracy in our match-to-sample task as a measure of visual speech processing difficulty across the 3 experimental conditions; (2) fixation patterns to the eyes and mouth (in terms of dwell time) as participants made linguistic judgements from visual speech; (3) latency to shift one’s eyes to the mouth at the beginning of the silent video; and (4) the overall patterns of saccades across critical portions of the trial. For all these four analyses, we excluded those trials where responses were made before the start of the first critical word that could differentiate the two sentences in each trial, which consisted of 5.96% of the total trials.

All statistical analyses were conducted using mixed-effects models in R (R Core Team) using the lme4 package (version 1.1–7) [35]. The random effects structure was specified in a maximal fashion for random slopes [36], with participant as the only random intercept: An items-intercept was not possible because only a subset of the sentences for each topic was presented within any individual participant for counterbalancing purposes. If this maximal model did not converge, the random effects structure was then reduced, but not the fixed effects structure (which specified the most critical experimental variables). This reduction process was done iteratively, first by decorrelating random slopes and the intercept, and then by removing a random slope, and then restarting this process. The first random slope to be removed was a slope at the highest order (e.g., slopes for 3-way interactions, then slopes for two-way interactions, and then main effects). If there were multiple slopes at the same order, then the slope that resulted in the smallest reduction in AIC/BIC values was removed. If the lme4 package did not provide p-values (i.e., for linear mixed-effects models), significance testing for fixed effects were obtained from Wald chi-square tests, obtained using the car package for R [37]. Post-hoc testing of fixed effects with more than two levels was done using Bonferroni corrections for multiple comparisons, all analyzed using the emmeans package from R [38].

### Analysis #1: Behavioral data

**Fig 2:**
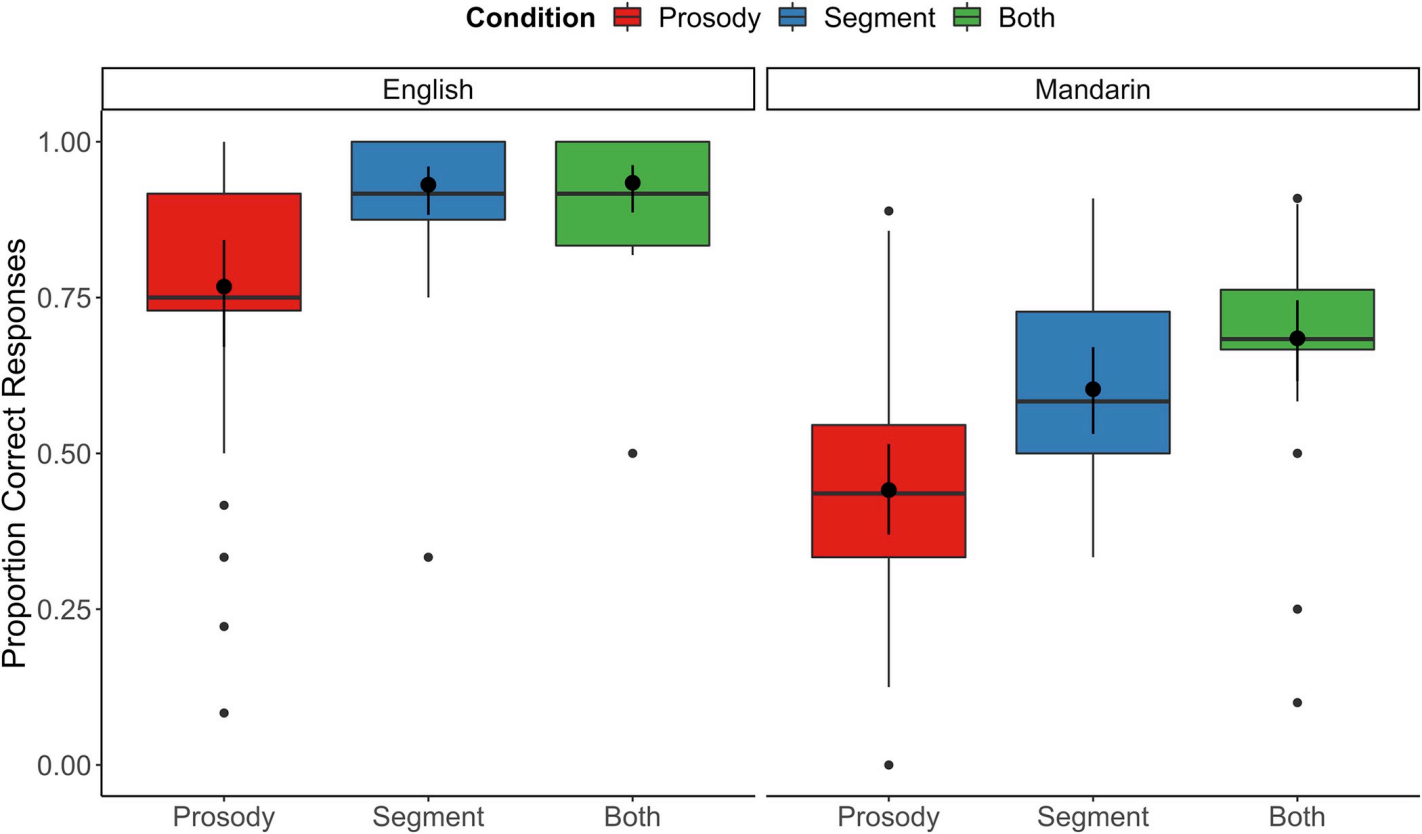
Proportion correct responding is displayed by language and by condition. Boxplots illustrate raw data, and the overlaid black dots and bars (95% *CI*s) indicate estimated means from the model.

For accuracy, trial responses were coded as either correct or incorrect (1 or 0, respectively), which was defined as whether the response (*left*, *right*, or *same auditory sentences*) matched the video stimulus. These responses were entered into a mixed-effects logistic regression with fixed effects of condition (Prosody, Segments, or Both), language (English or Mandarin), and their interaction. The most complex model that would converge had a random slope for language, and a random intercept for participant. Overall results suggested a marginally significant interaction between condition and language, *χ*^*2*^(3) *=* 5.84, *p* = .05, a significant main effect of language, *χ*^*2*^(1) *=* 79.18, *p* < .001, and a significant main effect of condition, *χ*^*2*^(1) *=* 68.77, *p* < .001. As shown in Fig 2, the effect of language is quite straightforward: Task accuracy was unsurprisingly higher in the native language (English) compared to an unfamiliar language (Mandarin).

As shown in Table 2, post-hoc tests of the estimated marginal means for the effect of the interaction revealed a similar pattern for both English and Mandarin trials. First, the odds ratio for the *Segment* compared to the *Prosody* condition was positive, indicating a significantly higher likelihood of being accurate in the *Segment* compared to *Prosody* conditions. This holds true when comparing *Both* and *Prosody* conditions. In addition, there were no significant differences between *Both* and *Segment* condition, indicating that the former was as accurate as the latter. Together, this suggests that performance in the *Prosody* condition was more difficult than in the *Segment* condition for both languages, while *Segment* condition and *Both* conditions have similar difficulty.

**Table 2:** Post-hoc analysis of the interaction between and language and condition.

Contrast		Odds ratio	*SE*	z-value	*p*
English	Prosody-Segment	-1.41	.26	-5.39	< .0001*
	Prosody-Both	-1.46	.27	-5.44	< .0001*
	Segment-Both	-.05	.40	-.16	1.00
Mandarin	Prosody-Segment	-.65	.18	-3.61	.005*
	Prosody-Both	-1.01	.19	-5.46	< .0001*
	Segment-Both	-.36	.18	-1.95	.76

Note. Post-hoc results are transformed from the mixed-effects logistic regression to odds ratios for ease of interpretation, done using the emmeans package in R (with Bonferroni corrections for multiple comparisons). Significant effects (*α* = .05) are in bold; very significant effects (*α* = .01) are asterisked.

In addition, we also analyzed participants’ mean response time by accuracy, condition and language. Response time was calculated by dividing their reaction time by the duration of the video presented in each trial, since each video clip has a different length. The descriptive results are shown in Table 3, which indicate participants at least needed to process an average of 77% of the stimulus sentence before they could make a response:

**Table 3:** Mean response time (in percentage) by condition and language.

		% Correct Trials	Correct RTs	% Incorrect Trials	Incorrect RTs
English	Prosody	73.94%	.83	26.06%	.90
	Segment	90.32%	.77	10.71%	.87
	Both	90.74%	.78	9.26%	.94
Mandarin	Prosody	44.62%	.91	55.38%	.92
	Segment	60.22%	.93	39.78%	.92
	Both	68.01%	.93	31.99%	.90

In summary, the results indicate that detecting visual differences between two auditory sentences was most difficult when these sentences differed only in prosody, and that this held true when scanning for linguistic information in the native (English) and non-native (Mandarin) languages. Specifically, prosodic differences were also more challenging than the detection of segmental differences, and the detection of both segmental and prosodic differences together. These results suggest that prosodic differences between auditory sentences were hardest to detect in our task.

### Analysis #2: Averaged fixation time to the eyes and mouth

The next question of interest was how participants scanned the silent face when searching for visual speech information. Specifically, we examined the proportion of dwell time to the eyes and mouth, and asked whether accuracy in the task (correct or incorrect response in a trial), the language of stimuli presentation (English or Mandarin), or experimental conditions (Segments, Prosody, Both) influenced these patterns of visual scanning. Eye and mouth looking were calculated using an Eyes-Mouth Index, which followed prior work [8, 29]. First, the proportion of time fixating to the Eyes and Mouth areas of interest (AOIs) were calculated relative to total looking time to the whole face within each trial. These numbers thus normalized across differences in total looking across individual trials. Second, the Eyes-Mouth Index was calculated by subtracting a difference score for each trial: the proportion gaze in the Eyes AOI–the proportion of gaze in the Mouth AOI. The formula is displayed as follows:

EMI=dwelltime(eyes)/dwelltime(face)‐dwelltime(mouth)/dwelltime(face)

Positive index scores (0 to 1) indicated more dwell time to the Eyes AOI, while negative scores (-1 to 0) indicate more dwell time to the Mouth AOI. So that just the visual scanning specifically linked to task performance was considered, these fixation proportions were calculated over the time window from video start until the response was made. Thus, for trials where the response came before the silent video ended, only dwell time in the pre-response window was considered. For trials where the response came after the entire silent video was played, all Eyes and Mouth AOI data were considered.

A subsequent analysis was then conducted. *T*rial-based Eyes-Mouth Index calculations were entered into a linear mixed-effects model as the dependent variable.

**Fig 3:**
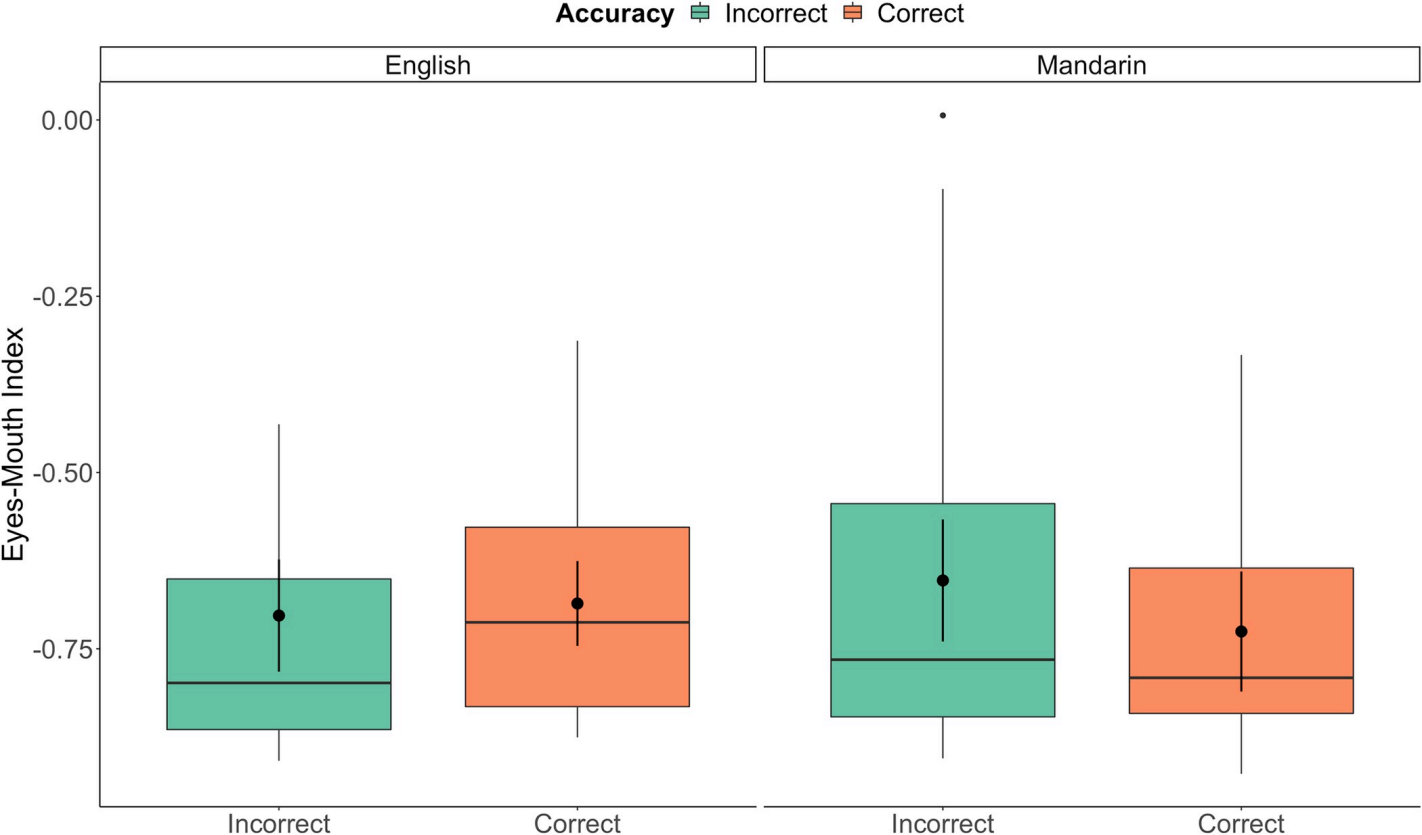
Looking to the eyes and mouth. Positive scores (0 to +1) indicated more looking to the Eyes AOI, while negative scores (0 to -1) indicate more looking to the Mouth AOI. The language * accuracy interaction in the Experimental condition is illustrated by the box plots. Overlaid black dots and bars (95% *CI*s) indicate estimated means from the model, with box plots showing raw data distributions.

Results from the analysis of experimental trials are shown in Table 4. In brief, there was a significant main effect of accuracy, as well as a significant interaction involving language and accuracy (shown in Fig 3).

**Table 4:** Effects from the model in the experimental analysis: Eye-mouth looking from averaged fixations.

Fixed Effects	Chi-square	*df*	p-value
Condition	2.10	2	.35
Language	.20	1	.66
**Accuracy**	**4.79**	**1**	.03*
Condition * Language	.25	2	.88
Condition * Accuracy	5.35	2	.07
Language * Accuracy	**9.06**	**1**	.003*
Condition * Language * Accuracy	4.26	2	.12

Note. Bold indicates significant effects (α = .05), with asterisks indicating significant effects.

Post-hoc pairwise analysis using the emmeans() function on this interaction of language and accuracy (as shown in Fig 3) suggested that there was no effect of accuracy in visual scanning when doing this task in English, *p* = .56. However, in Mandarin, there was longer looking to the mouth indicated by a decrease in the Eyes-Mouth Index, *M* = .07, *SE =* .02, *p* = .0004, when responses were correct compared to incorrect.

The interaction between language and accuracy further suggests that when hearing an unfamiliar language, the successful detection of visual speech cues was associated with a greater difference in reliance on the mouth v.s. the eyes. We interpret this effect as showing that participants were searching for visual cues from the mouth area to confirm their initial judgements about auditory information (i.e., their memory about what was different between the sentences). Importantly, this distinction was not found in trials where subjects were exposed to their native language. That is, when the language was a familiar one, there was not significantly more focus on the mouth area on correct versus incorrect.

### Analysis #3: Saccade amplitude

The last question we investigated is whether visual scanning across the relevant portion of the silent video differed across trials in the different conditions and language. This scanning measure is a way of capturing global scanning strategies, as saccade amplitudes can show the degree to which visual scanning is diffuse perhaps looking at larger regions of the mouth or eyes, or whether scanning was more focal, and concentrated at a specific area. Greater saccade amplitudes indicated more general, diffuse scanning whereas a smaller one indicates a more focused fixation. We measured the current amplitude of each individual saccade in each trial made by each participant. Our statistical models asked whether saccade amplitudes were affected by accuracy, the language of stimuli presentation, or experimental condition.

**Fig 4:**
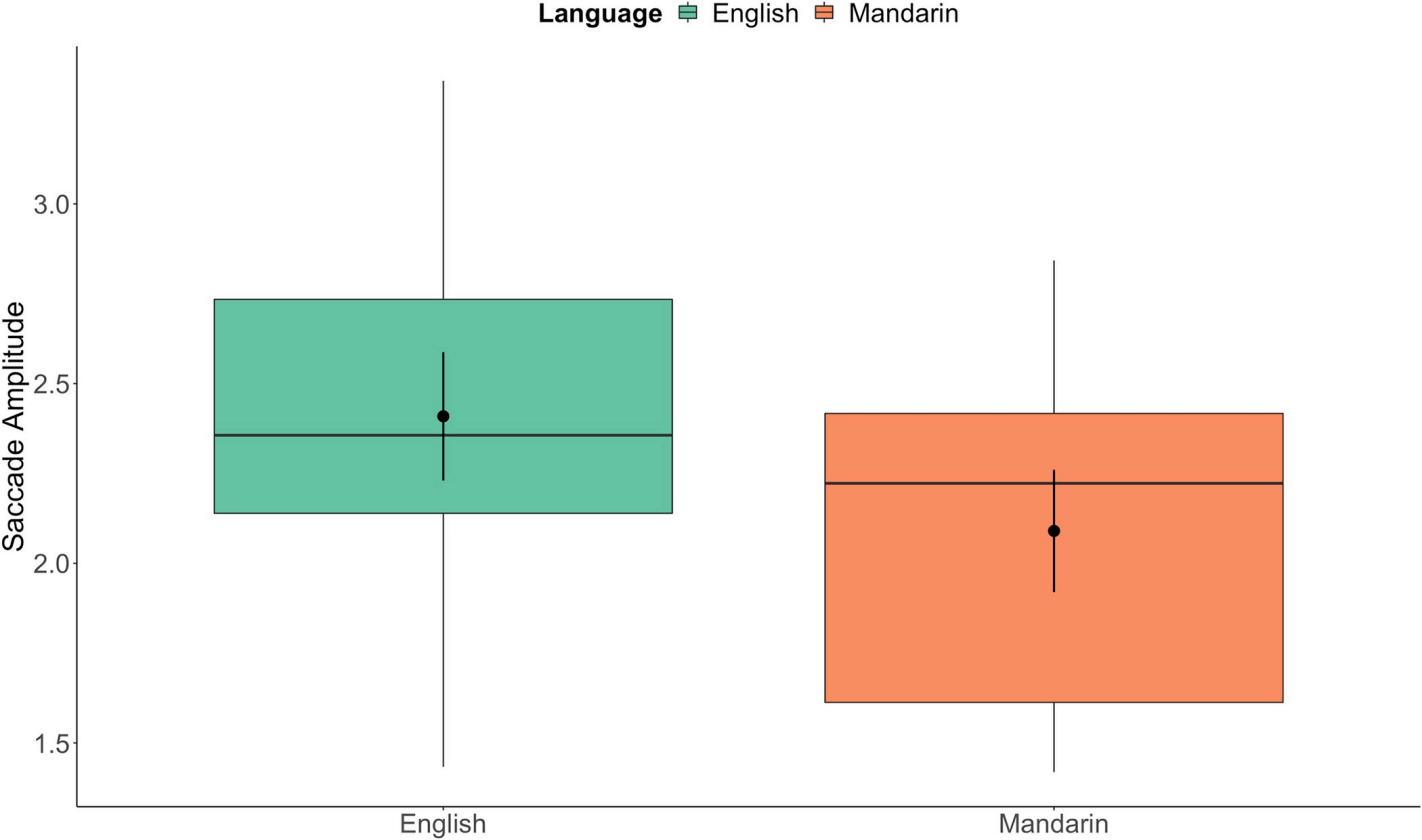
Saccade amplitude. The main effect of language in the Experimental condition is illustrated by the box plots. Overlaid black dots and bars (95% *CI*s) indicate estimated means from the model, with box plots showing raw data distributions.

Results from the analysis of experimental trials are also shown in Table 5. Here, there was only one significant effect, which is a main effect of language. Unlike the results of fixation proportions, there was no significant interaction involving language and accuracy (shown in Fig 4). Across all the trials, the saccade amplitude in English trials was significantly greater than that in Mandarin trials (as shown in Fig 4).

**Table 5:** Effects from the model in the experimental analysis: Saccade amplitudes.

	Chi-square	*df*	*p*-value
Condition	0.28	2	0.87
**Language**	**42.19**	1	<0.001**
Accuracy	0.01	1	0.92
Condition * Language	0.41	2	0.82
Condition * Accuracy	0.43	2	0.81
Language * Accuracy	0.22	1	0.64
Language * Accuracy * Condition	4.69	2	0.10

Note. Bold indicates significant effects (α = .05), with asterisks indicating significant effects (α = .05).

In summary, the result suggests larger saccades were made in English trials than in Mandarin trials when the participants made both correct and incorrect responses. This may reflect a similar pattern showed in the first analysis of fixation proportion to the eyes or mouth, but from a different angle. That is, these results may be due to participants’ different visual scanning behaviour in search of speech information when listening to a native versus a non-native language, that perhaps they tended to move their eye gaze between facial features more frequently for their native language than for the non-native language.

### Analysis #4: Fixation latencies

**Fig 5:**
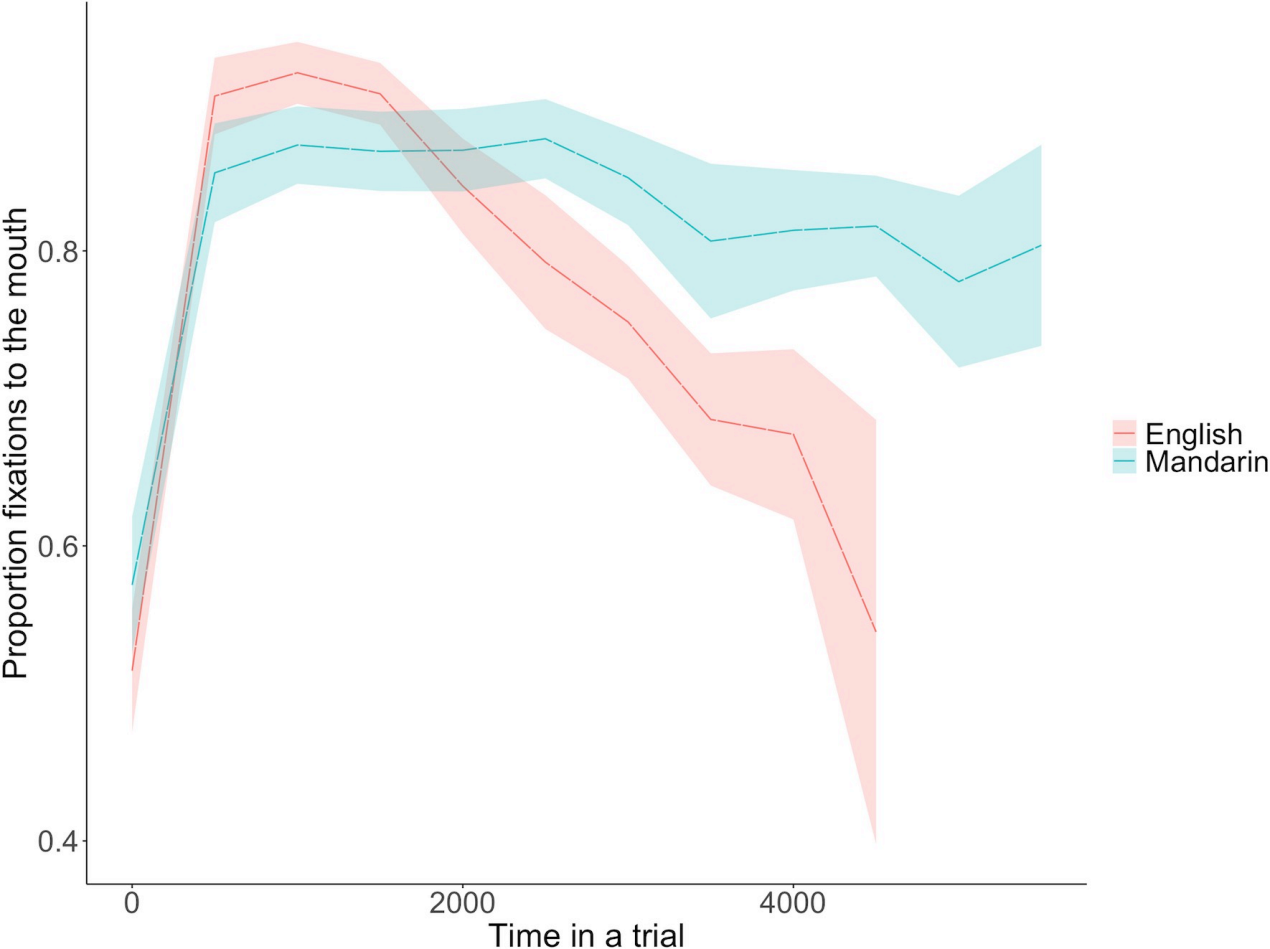
General looking patterns from all trials in different language conditions. The x-axis represents the time since the trial started and the y-axis represents the proportion looking to the mouth relative to the sum of looking to both mouth and eyes.

As can be seen in Fig 5, participants necessarily began looking at the AOI to the eyes when the video appeared, and then at some point shifted their gaze to the mouth. Visual inspection of these looking patterns in English and Mandarin trials generally show that there was a steeper slope of the initial shift to the mouth in English, indicating shifting to the mouth was faster in English. Here we investigated participants’ time to initiate their first look to the mouth, or the speed with which participants decided to begin a search for visual information from the mouth. We calculated this more precisely by examining the duration of time it took for participants to launch their first eye gaze to the mouth from the beginning of the video, and again investigated any potential influences of accuracy, the language of stimuli presentation, and condition. Similar to the previous analyses, all the trials were analyzed separately following the same method.

Model results from the analysis of experimental trials are shown in Table 6. Here, there were two significant effects, which are detailed in Table 6: A significant main effect of language, and a significant interaction involving condition and language.

**Table 6:** Effects from the model in the experimental analysis: Fixation latencies.

	Chi-square	*df*	*p*-value
Condition	4.00	2	.14
Language	3.54	1	.06
**Accuracy**	**4.80**	1	.03*
Condition * Language	**8.31**	2	.02*
Condition * Accuracy	5.65	2	.06
Language * Accuracy	.78	1	.38
Language * Accuracy * Condition	.15	2	.93

Note. Bold indicates significant effects (α = .05).

**Fig 6:**
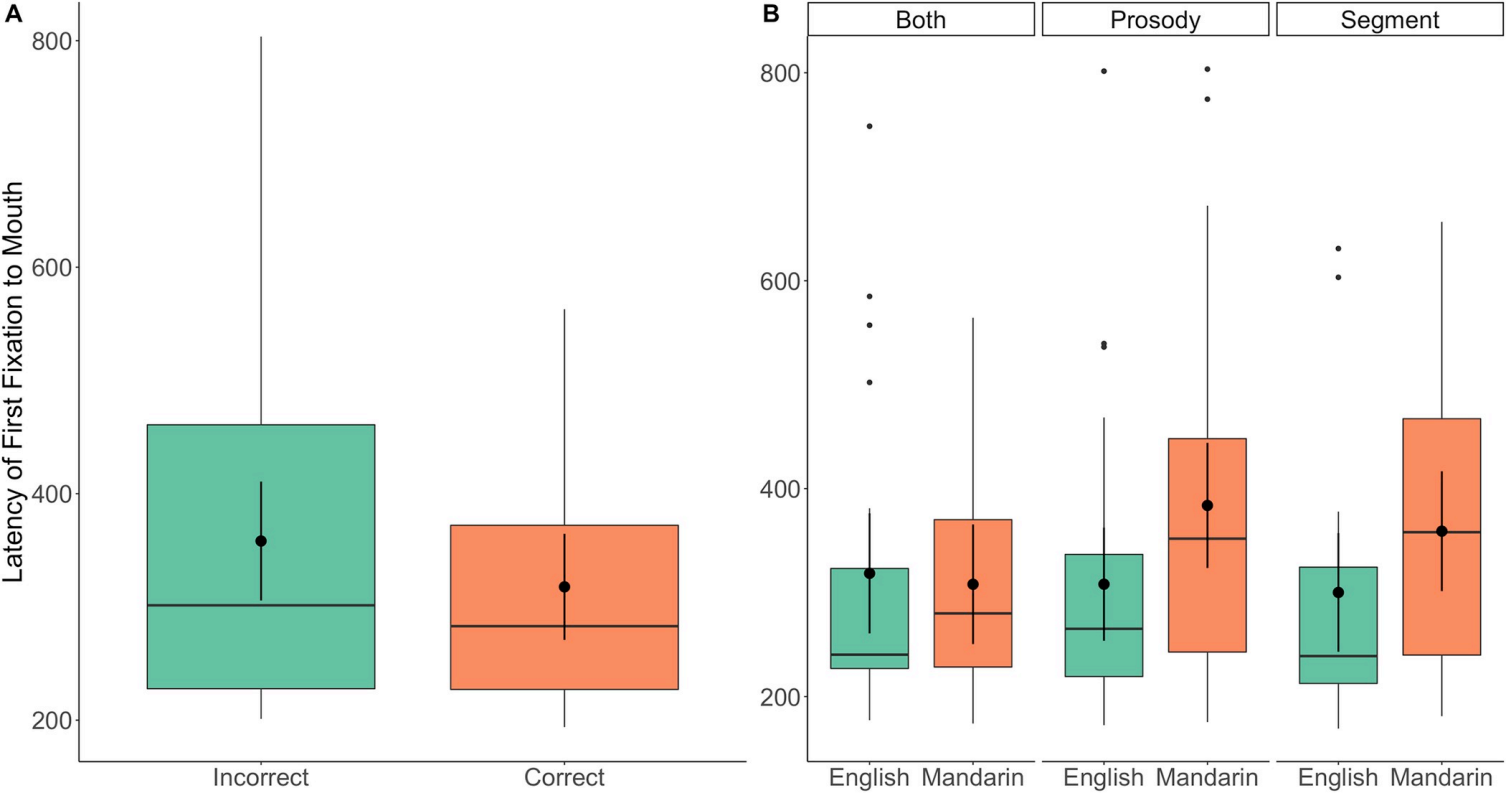
First fixation: duration of time from the beginning of a video to the first shift to the mouth. The main effect of accuracy is shown in (A), while (B) illustrates the Language * Condition interaction for the experimental conditions. Overlaid black dots and bars (95% *CI*s) indicate estimated means from the model, with box plots showing raw data distributions.

The main effect of accuracy and the interaction between condition and language are shown in Fig 6. Results indicated that there was a significant delay of 68.67 ms for launching the initial shift in incorrect trials than correct trials across both languages and all conditions. Crucially, there was a significant effect of language in *Prosody* condition, where there was a 75.02 ms (*M* = -70.8) slower initial shift to the mouth in Mandarin trials than English trials, *p* = .02. However, in the *Segments* and *Both* condition, there was no significant difference in the latency of the initial shift between the two languages, *p* = .10 and *p* = .55, respectively.

In summary, fixation latencies are associated with the speed of participants’ active search about visual speech information from the mouth, which may carry distinctive linguistic information. Successful match between auditory information and visual cues appeared to be associated with faster initial shift to the mouth. More importantly, when there were only prosodic differences, participants’ latencies to begin searching the mouth area were significantly delayed when they received input in the unfamiliar language compared to the familiar language. This may indicate that participants were not as sure in Mandarin trials as in English trials about where to find prosodic information in a face, or alternatively, it may indicate global delays in processing prosodic visual speech in Mandarin. However, when only segmental differences were presented, or when both segmental and prosodic differences were salient, there was no difference between latency to look to mouth across languages.

We interpret these results as showing that, when participants successfully detected the auditory differences and eventually matched them with the correct visual information, they also tended to exhibit greater certainty of searching the mouth area, regardless of these differences being segmental and/or prosodic in a familiar or unfamiliar language. In addition, when participants have an easier time detecting differences from the audio sentences in the *Both* and *Segments* condition, they were perhaps more expecting that useful cues might be embedded in mouth movements regardless of which language they were listening to. However, when the task became more difficult in the *Prosody* condition, they were slower to look to the mouth in the Mandarin trials, relative to the English ones. Thus, when encountering an unfamiliar language, participants were perhaps not as confident about the role of mouth in providing useful information, suggesting that participants’ expectations that certain speech cues are encoded in mouth movements might have been reflected in latencies to search that area of the face.

## Discussion

In the current study, we found that there was a greater difference of fixations to the mouth vs. the eyes in the correct trials over incorrect trials, but only when processing the non-native language. Additionally, the saccade amplitude in the non-native trials was smaller than in the native language trials. These two aspects of scanning behavior together suggest that more focal and longer gaze at the mouth is associated with extraction of useful cues that facilitate speech processing, particularly when listeners did not have any linguistic knowledge of the language. In addition, the result of fixation latency analysis shows that listeners attended to the mouth more slowly when processing prosodic information in their non-native language (relative to segmental information).

Earlier, we proposed research questions about whether listeners’ scanning patterns as they were searching for different linguistic information would differ when processing a native versus non-native language, and whether any particular scanning behavior would be associated with more accurate detection of speech. Below, we will firstly discuss why different scanning behavior was elicited when processing native versus non-native languages (for the two analyses of fixation proportions and saccade amplitude). Then, we will discuss why we only found minimal effects of a search for segmental versus prosodic cues. Finally, we discuss the interaction of language and phonetic information type: Why listeners would look to the mouth with a longer latency when processing prosodic (versus segmental) information, but only in the non-native language.

### Native versus non-native language processing

Overall, our analyses of visual scanning suggest that, when processing a non-native language, there was more focal and longer search of the mouth area. More specifically, more dwell time to the mouth over eyes predicted more successful detection of segmental and/or prosodic information. This measure was less pronounced when processing the native language (although more mouth fixations were found, the difference between the mouth and eyes was smaller), which suggests that mouth-looking played a more important role in achieving an accurate match between auditory memory and relevant facial cues when processing was harder.

This echoes prior results from [8, 29], where there were overall more fixations to the mouth than to the eyes when actively encoding the speech information from a non-native language compared to processing a native language. Prior work has already shown that having access to visual cues is useful when auditory resources are limited, such as in noisy, degraded or semantically and syntactically complex speech [2–5, 7, 39]. Processing a non-native language in these cases, could also be quite challenging, since access to both higher-level lexical and phonological knowledge are no longer available. The current study not only confirms that perceivers solicit aid from mouth gestures when the auditory task is challenging, but also complements this prior work by further suggesting an association between scanning behaviour at the mouth and the successful matching of speech information with auditory memory of sounds. Note that our stimuli differ from those in the previous study by [26], as their sentences consisted of two words, while ours were longer, naturally articulated sentences that required greater cognitive load to focus on segmental and suprasegmental differences on two syllables. Participants in our study needed to identify key differences, and keep them in mind until they found a visual match for the auditory cues: We ensured that the first critical difference lay within the first three words for all auditory stimuli, which does not exceed adults’ mean working memory span of 6.02 items for word strings [40]. Therefore, our interpretation of the result that participants made more fixations to the mouth in order to find out the correct visual correlates in Mandarin trials, is that when listening to English trials, participants compared visual speech cues to internal models of English phonemes, words, and phrases, thus relying less on the extraction of bottom-up visual cues.

To be more specific, real-time word processing in a native language may take place more equitably in both bottom-up and top-down manners [41, 42], compared to processing a non-native language. Thus, having access to higher-level resources, like lexical and phonological information, might enhance the activation of the acoustic and articulatory features of certain phonemes [43, 44]. When listeners were searching the English-speaking face, then, they may have been targeting the critical cues faster, being able to more accurately and promptly move their eye gaze away from the mouth as soon as they successfully matched the facial cues with the first acoustic difference stored in their memory. This may be able to explain why, in both [29] and our current study, participants’ accuracy reached a ceiling effect for English stimuli whether they successfully matched critical visual cues with auditory differences or not. That is, there was not as much a need to allocate more attention to the mouth area in cases of lower cognitive load.

On the contrary, when listening to Mandarin trials, it may have been much more difficult for them to encode these linguistic units, and thus their visual search was necessarily guided by a visual match to their *acoustic* memory traces of the sentences, as access to higher-level phonemic and lexical knowledge was not possible without any knowledge of Mandarin. Therefore, when watching the video in Mandarin trials, participants may have required more time to match visual cues purely onto their acoustic memory trace, perhaps needing multiple distinctive mouth gestures embedded in different words to validate their initial judgement and then be confident enough to make a decision, which may have also resulted in delayed, longer and more focused looking to the mouth. This assumption is indeed verified by the general looking pattern shown in Fig 5. After the initial shift to the mouth, there was more constant gaze at the mouth in Mandarin trials, whereas listeners’ eye gaze quickly shifted back to the eyes in English trials sooner (presumably after they found the critical differentiating cues). Unlike [29], our participants did not reach a ceiling effect for the accuracy in the unfamiliar language (Mandarin, in our case). This may have been because our auditory sentences were longer than [29] study, and thus more difficult to process. However, both their and our results indicate that longer and more focal looking to the mouth area is a necessary condition for successfully decoding an unfamiliar language at the acoustic level.

These results were reflected in the main effects seen in all our analyses. However, these language-based differences in task strategies may also have manifested in several ways beyond longer, more focused, and slower mouth-looking for non-native stimuli. Note that in Fig 3, there was larger variation in where participants scanned when making incorrect responses on Mandarin trials than English trials, suggesting alternative strategies when one is unable to successfully use mouth information to match auditory memory traces. It is also possible that some listeners failed to accurately differentiate the two auditory sentences in the first place at either segmental or prosodic level, and when they were watching the silent talking face, they would search the whole face to find whatever was accessible to them. Alternatively, upon failing to detect the differences, participants may also have made less effort to searching the mouth, knowing that it would be useless even if they did have visual speech information from mouth.

### Phonetic information type: Segmental versus prosodic information

In most analyses, results showed minimal effects of segmental versus prosodic information (i.e., we found no type effects in either the fixation proportion or saccade amplitude analyses, and no main effect of type in the latency analysis). This seems to argue against some prior results, including [26–28, 45], where the presence of facial cues from the upper part of the face (or from facial movement in the forehead, eye-brows, or whole head) significantly facilitated the decoding of prosodic information. However, different types of prosodic information depend on the cues from the upper part of the face to different extents. For example, for prosodic variation at a more global level like intonation, in [27], it was kinetic head motion that was found to be highly correlated with F0 and amplitude variance over a whole sentence, and participants could identify more syllables in a sentence under a noisy condition with the help of the prosodic information indicated by head movement. In a parallel fashion, [26] found that intonation patterns (a question or statement) versus word-level stress (sentential prominence) resulted in different proportions of eye gaze frequency towards the upper part of the face. Indeed, the present study paralleled that result in showing no significant (but rather just a marginal difference) in looking to the top and bottom of a face for prosodic information at a more local level, i.e. word-level sentential stress cues.

There may be two additional reasons contributing to our null result. First, in our experiment design, participants needed to firstly identify any kind of differences auditorily when lisntening to the two sentences. Prosodic differences may have been more difficult for them to identify than segmental differences, as indicated by the lowest behavioral accuracy rate in the *Prosody* condition among all the conditions. Looking to the mouth area may have been, by default, the only reliable source of linguistic information without proper auditory encoding (and this may have been especially be true for the Mandarin trials). For trials in the *Both* condition, participants may have paid more attention to segmental differences, resulting in no differences from the *Segment* condition. Another possibility may be that the mouth also carries cues for prosody, especially for sentential stress. [46] found that listeners could reliably match prosodic cues encoded by auditory item and visual token no matter whether the upper or lower area of the face was shown. Furthermore, other researchers [34] found that, among various facial cues including eye brow, head and lip movement, it was chin displacement for an opening gesture that contributed the most to the perception of phrasal stress. Likewise, [47] also suggested that the lip area and jaw opening facilitated the most successful differentiation of phrasally stressed items in French. Although in the current study, we did not measure the acutal visual cues exerted by the bilingual speaker and then correlate them with perceivers’ scanning behavior, the parellel looking patterns found for both segmental and prosodic information indicates that perceivers at least had a similar expectation for useful cues being primarily concentrated in the mouth area for processing prosodic information (specifically sentential stress in this case), just as they did for segmental information, no matter whether the potential cues eventually faciliated successful decoding or not, causing no differences in our phonetic information type manipulation.

### Interaction between language and phonetic information type

Another major result was that there was a longer latency of shifting eye gaze to the mouth in Mandarin trials than in English trials, but only when perceivers were processing prosodic information. This fixation latency reflects participants’ awareness of whether the mouth could carry the critical cues for the auditory differences they just detected. In [33], the latency of the first fixation to the subtitles on the screen reflected participants’ reliance on these subtitles to comprehend the speech and visual scenes in the movie. There was greater latency when the subtitles were in a foreign language or when the foreign subtitles were in a two-line form, which reflects the need to seek complementary help from the subtitles to achieve better comprehension. Similarly, in the current study, the delayed shift in the *Prosody* condition of Mandarin trials (compared to English), may be another piece of evidence for the assumption discussed previously, which was that prosodic differences (especially in a non-native language,) are quite difficult for listeners to detect. Failing to detect prosodic differences may have resulted in an accompanying hesitation in searching the mouth for useful cues in the first place.

Note that the difference between the fixation latencies to the mouth when processing the two languages disappears in the *Segments* and *Both* condition. This suggests that segmental differences in Mandarin were easier to detect, and that perceivers were confident about searching the mouth for useful cues. In the *Both* condition, even in the non-native language, there may have been more opportunities for perceivers to encode critical information from different linguistic domains, which may have resulted in the same degree of readiness in the non-native language to fixate the mouth as they had for their native language.

The delay of looking to the mouth in the current results does not seem to indicate that listeners were aware of the auditory differences in the prosodic domain, and deliberately chose to stay at the eyes instead. If it was really the case, there would have been a by-condition difference among the English trials as well, where the fixation latency was longer when processing prosodic differences than when processing segmental differences. Moreover, fixation latency not only seems to be an indicator of the ease of auditory detection, it was also associated with success in our task across both languages and all information types, since accuracy appeared to be a significant main effect. However, initial correct identifications of the auditory differences may be differently associated with gaze strategies during the visual search in English and Mandarin trials. Even when listeners succeeded in detecting the auditory differences in Mandarin segments, or at least were confident about their detection to the same extent as English segments, they still needed to exhibit longer and more constant gaze at the mouth to ensure an accurate visual search later. For Mandarin prosody, even when listeners found it more difficult to auditorily detect any differences (reflected by a delayed fixation latency), they still focused more on the mouth as if it was a default strategy (reflected by more mouth looking in Mandarin trials when correct responses were made without by-condition differences).

## Concluding remarks

This study investigated listeners’ visual scanning of a talking face when processing segmental and prosodic information in their native versus non-native language. Previous studies have found that more fixations were allocated to the mouth when processing speech in more challenging situations, such as when perceiving a non-native language [29, 30]. Other work shows that there is more looking to the eyes, or more diffuse looking to the whole face, when decoding prosodic information compared to segmental information [26–28]. Compared to studies that investigated these two factors separately and did not examine language and phonetic information type in the same experiment, the current study further asked how these two phenomena interact, and whether different visual cues could indeed facilitate decoding speech information in a native versus a non-native language, which confirmed our hypothesis that there would be more accentuation of looking to the mouth area when processing non-native speech. The results showed that when processing a non-native language, perceivers displayed more fixations and constant eye gaze at the mouth area and longer fixation time was strongly associated with the successful detection of segmental and prosodic information. In addition, there was a longer latency of eye gaze shift to the mouth in Mandarin trials than in English trials when perceivers were processing prosodic information, corroborating our hypothesis that a delayed shift to the mouth area was linked to a more difficult match between any auditory traces in memory and gestural information from the mouth in auditorily more challenging situations, such as processing prosodic information and/or non-native speech.

The current study points to a number of future directions. First, our current study only investigated one prosodic type, contrastive focus, which in previous studies [26–28] has shown mixed results in terms of whether perceivers fixated more the mouth or eyes. Future studies may also explore other dimensions of prosody, such as phrase-level stress as well as sentence-level intonation, while keeping the general structure of the experiment to see whether there would be a difference of saccade amplitude as well as fixations to the eyes, and whether there would be an interaction between language and the type of linguistic information targeted in the task. Second, although the bilingual talker in our experiment was required to retain a neutral face while producing the stimuli, we cannot guarantee that there were not any language- or culture-induced differences of facial features at all when speaking English versus Mandarin (e.g., there might be more eye-brow raising when speaking English). However, we were interested in using unedited, naturalistic stimuli in this study (similar to prior work, like [29]), and future analyses could try to link talker movements to the perceivers’ eye movements like in some of previous studies [27, 28, 34, 47], as our data and analysis are open. Future studies may thus try to record the amount of visual cues such as head movements when the talker is speaking a familiar versus an unfamiliar language, and/or producing segmental versus prosodic contrasts. They could then correlate these visual cues with listeners’ ability to perceive linguistic units on these dimensions. Third, other than the factor of language familiarity (nativeness), the availability of auditory and visual cues may be different in English and Mandarin. Thus, the factor of language difference may also contribute to the current looking patterns. Future studies may include both native English and native Mandarin perceivers to examine potential interactive effects of “language” and “nativeness” differences. Finally, the current study mainly investigated the average fixations, saccade amplitude overall the whole trial. Future studies that wish to explore these effects in more fine-grained detail may also consider restricting the stimuli sentences to conduct a time-course analysis to investigate perceivers’ looking pattern when encountering the first critical word that differentiates the two sentences, and how it changes over time as information from the sentence is processed.

In conclusion, results revealed that there was an association between scanning behaviour (i.e., more gaze at the mouth) and the successful encoding of auditory speech. Moreover, this varied by language: When using native linguistic knowledge, English-speaking participants could identify linguistic differences more quickly and looked less (and less focally) at the mouth, whereas in Mandarin trials, these same participants required more information from the mouth. Additionally, the delayed latency in the *Prosody* condition of Mandarin trials relative to that of English trials may indicate participants’ difficulty in identifying prosodic information in a non-native language, and thus required more time to fixate the mouth. The current study thus extends previous research by elaborating listeners’ scanning behavior in real-time processing: In addition to exploring fixation duration at each facial area, we explored how listeners moved their eye gaze across different facial areas and how fast they attended to the mouth to search for critical information. The current study also complements previous studies by concentrating on how scanning patterns differ when processing different speech information in a non-native language and by associating particular scanning patterns with efficacious comprehension of naturalistic speech.

## Supporting information

S1 Appendix Stimulus sentences in the eye-tracking experiment. (ZIP)
