# Supplementary material for: Visual scanning patterns of a talking face when evaluating phonetic information in a native and non-native language
Source: PLoS One. 2024 May 28;19(5):e0304150. doi: 10.1371/journal.pone.0304150 (PMC11132507; doi:10.1371/journal.pone.0304150)
Supplement: S1 Appendix — (ZIP) [file pone.0304150.s001.zip › Appendix/Appendix.docx]

**Appendix**

| Full set | Condition |
| --- | --- |
| **Reference Sentence:** No, **JESS** found her **PLAIN** dress for **KYLA’S** wedding.  No, **JESS** found her **PLAIN** dress for **KYLA’S** wedding. | Baseline |
| No, Jess **FOUND** her plain **DRESS** for Kyla’s **WEDDING**. | Prosody |
| No, **ANNE** found her **BLUE** dress for **JIMMY’S** wedding. | Segments |
| No, Anne **FOUND** her blue **DRESS** for Jimmy’s **WEDDING**. | Both |
| **Reference Sentence:** No, **TODAY** Jimmy **COOKED** himself a large **BURGER.**  No, **TODAY** Jimmy **COOKED** himself a large **BURGER.**  No, Today **JIMMY** cooked **HIMSELF** a **LARGE** burger.  No, **THIS** **WEEK** Jimmy **PAID** himself a large **BONUS**.  No, This week **JIMMY** paid **HIMSELF** a **LARGE** bonus. | Baseline  Prosody  Segments  Both |
| Reference Sentence: Actually, Carla **HOPED** to climb the **STEEP** trail on the **HILLSIDE**.  Actually, Carla **HOPED** to climb the **STEEP** trail on the **HILLSIDE**.  Actually, **CARLA** hoped to **CLIMB** the steep **TRAIL** on the hillside.  Actually, Carla **WISHED** to climb the **MUDDY** trail on the **FIELD**.  Actually, **CARLA** wished to **CLIMB** the muddy **TRAIL** on the field. | Baseline  Prosody  Segments  Both |
| **Reference Sentence:** Actually, **ANDREW** wanted to **CANOE** under the **ARCHWAY** tonight.  Actually, **ANDREW** wanted to **CANOE** under the **ARCHWAY** tonight.  Actually, Andrew **WANTED** to canoe **UNDER** the archway **TONIGHT**.  Actually, **PHYLLI**S wanted to **RELAX** under the **AWNING** tonight.  Actually, Phyllis **WANTED** to relax **UNDER** the awning **TONIGHT**. | Baseline  Prosody  Segments  Both |
| **Reference Sentence:** No, **MICHELLE** wants a **BLACK** wooden table for **HER** house.  No, **MICHELLE** wants a **BLACK** wooden table for **HER** house.  No, Michelle **WANTS** a black **WOODEN** table for her **HOUSE**.  No, **JAYDA** wants a **WHITE** wooden table for **MY** house.  No, Jayda **WANTS** a white **WOODEN** table for my **HOUSE**. | Baseline  Prosody  Segments  Both |
| **Reference Sentence:** No, **JESSIE** wants to see a classic **PLAY** with Ted and **RUTH**.  No, **JESSIE** wants to see a classic **PLAY** with Ted and **RUTH**.  No, Jessie **WANTS** to see a **CLASSIC** play with **TED** and Ruth.  No, **JOEY** wants to see a classic **SHOW** with Ted and **PAUL**.  No, Joey **WANTS** to see a **CLASSIC** show with **TED** and Paul. | Baseline  Prosody  Segments  Both |
| **Reference Sentence:** 不，小**李**想给 他的大**姐**买 只 **红**色的 手表。  不，小**李**想给 他的大**姐**买 只 **红**色的 手表。  不，小李**想**给他的大姐**买**只红色的**手**表。  不，小**马**想给他的大**嫂**买只 **银**色的手表。  不，小马**想**给他的大嫂**买**只银色的**手**表。 | Baseline  Prosody  Segments  Both |
| **Reference Sentence:** 不，小**陈**想穿**白**的裙子去小**花**的婚礼。  不，小**陈**想穿**白**的裙子去小**花**的婚礼。  不，小陈**想**穿白的**裙**子去小花的**婚**礼。  不，小**王**想穿**蓝**的裙子去小**冬**的婚礼。  不，小王**想**穿蓝的**裙**子去小冬的**婚**礼。 | Baseline  Prosody  Segments  Both |
| **Reference Sentence:** 不，**上**周末马克在教室**换** 了一袋**糖**。  不，**上**周末马克在教室**换** 了一袋**糖**。  不，上周末**马**克在教**室**换了一**袋**糖。  不，**这**周末马克 在教室**背**了一个**词**。  不，这周末**马**克在教**室**背了一**个**词。 | Baseline  Prosody  Segments  Both |
| **Reference Sentence:** 事实上，**老**王下班后想去**走**陡的**山**路。  事实上，**老**王下班后想去**走**陡的**山**路  事实上，老王 下**班**后想去走**陡**的山**路**。  事实上，**沈**王下班后想去**跑**陡的**公**路。  事实上，沈王下**班**后想去跑**陡**的公**路**。 | Baseline  Prosody  Segments  Both |
| **Reference Sentence:** 不，丁当想在**客**厅里摆**棕**色的木**桌**。  不，丁当想在**客**厅里摆**棕**色的木**桌**。  不，丁**当**想在客厅里**摆**棕色的**木**桌。  不，丁当想在**饭**厅里摆**黑**色的木**雕**。  不，丁**当**想在饭厅里**摆**黑色的**木**雕。 | Baseline  Prosody  Segments  Both |
| **Reference Sentence:** 不，她是来自**西**方的**金**发新人**戏**剧演员  不，她是来自**西**方的**金**发新人**戏**剧演员  不，**她**是来自西方的金发**新**人戏剧**演**员。  不，她是来自**东**方的**棕**发新人**话**剧演员。  不，**她**是来自东方的棕发**新**人话剧**演**员。 | Baseline  Prosody  Segments  Both |
