# Supplementary material for: Visual scanning patterns of a talking face when evaluating phonetic information in a native and non-native language
Source: PLoS One. 2024 May 28;19(5):e0304150. doi: 10.1371/journal.pone.0304150 (PMC11132507; doi:10.1371/journal.pone.0304150)
Supplement: S1 Appendix — (ZIP) [file pone.0304150.s001.zip › Appendix/Appendix(English).docx]

**Appendix**

| Full set | Condition |
| --- | --- |
| **Reference Sentence:** No, **JESS** found her **PLAIN** dress for **KYLA’S** wedding.  No, **JESS** found her **PLAIN** dress for **KYLA’S** wedding. | Baseline |
| No, Jess **FOUND** her plain **DRESS** for Kyla’s **WEDDING**. | Prosody |
| No, **ANNE** found her **BLUE** dress for **JIMMY’S** wedding. | Segments |
| No, Anne **FOUND** her blue **DRESS** for Jimmy’s **WEDDING**. | Both |
| **Reference Sentence:** No, **TODAY** Jimmy **COOKED** himself a large **BURGER.**  No, **TODAY** Jimmy **COOKED** himself a large **BURGER.**  No, Today **JIMMY** cooked **HIMSELF** a **LARGE** burger.  No, **THIS** **WEEK** Jimmy **PAID** himself a large **BONUS**.  No, This week **JIMMY** paid **HIMSELF** a **LARGE** bonus. | Baseline  Prosody  Segments  Both |
| Reference Sentence: Actually, Carla **HOPED** to climb the **STEEP** trail on the **HILLSIDE**.  Actually, Carla **HOPED** to climb the **STEEP** trail on the **HILLSIDE**.  Actually, **CARLA** hoped to **CLIMB** the steep **TRAIL** on the hillside.  Actually, Carla **WISHED** to climb the **MUDDY** trail on the **FIELD**.  Actually, **CARLA** wished to **CLIMB** the muddy **TRAIL** on the field. | Baseline  Prosody  Segments  Both |
| **Reference Sentence:** Actually, **ANDREW** wanted to **CANOE** under the **ARCHWAY** tonight.  Actually, **ANDREW** wanted to **CANOE** under the **ARCHWAY** tonight.  Actually, Andrew **WANTED** to canoe **UNDER** the archway **TONIGHT**.  Actually, **PHYLLI**S wanted to **RELAX** under the **AWNING** tonight.  Actually, Phyllis **WANTED** to relax **UNDER** the awning **TONIGHT**. | Baseline  Prosody  Segments  Both |
| **Reference Sentence:** No, **MICHELLE** wants a **BLACK** wooden table for **HER** house.  No, **MICHELLE** wants a **BLACK** wooden table for **HER** house.  No, Michelle **WANTS** a black **WOODEN** table for her **HOUSE**.  No, **JAYDA** wants a **WHITE** wooden table for **MY** house.  No, Jayda **WANTS** a white **WOODEN** table for my **HOUSE**. | Baseline  Prosody  Segments  Both |
| **Reference Sentence:** No, **JESSIE** wants to see a classic **PLAY** with Ted and **RUTH**.  No, **JESSIE** wants to see a classic **PLAY** with Ted and **RUTH**.  No, Jessie **WANTS** to see a **CLASSIC** play with **TED** and Ruth.  No, **JOEY** wants to see a classic **SHOW** with Ted and **PAUL**.  No, Joey **WANTS** to see a **CLASSIC** show with **TED** and Paul. | Baseline  Prosody  Segments  Both |
| **Reference Sentence:** No，Xiao**li** wants to buy his elder **sister** a **red** watch.  No，Xiao**li** wants to buy his elder **sister** a **red** watch.  No，Xiaoli **wants** to **buy** his elder sister a red **watch**.  No，Xiao**ma** wants to buy his **sister-in-law** a **silver** watch.  No，Xiaoma **wants** to **buy** his sister-in-law a silver **watch**. | Baseline  Prosody  Segments  Both |
| **Reference Sentence:** No, Xiao**chen** wants to wear a **white** dress for Xiao**hua**’s wedding.  No, Xiao**chen** wants to wear a **white** dress for Xiao**hua**’s wedding.  No, Xiaochen **wants** to wear a white **dress** for Xiaohua’s **wedding**.  No, Xiao**wang** wants to wear a **blue** dress for Xiao**dong**’s wedding.  No, Xiaowang **wants** to wear a blue **dress** for Xiaodong’s **wedding**. | Baseline  Prosody  Segments  Both |
| **Reference Sentence:** No, **last** week Mark **exchanged** a bag of **candy** in the classroom.  No, **last** week Mark **exchanged** a bag of **candy** in the classroom.  No, last week **Mark** exchanged a **bag** of candy in the **classroom**.  No, **this** week Mark **memorized** a **word** in the classroom.  No, this week **Mark** memorized **a** word in the **classroom**. | Baseline  Prosody  Segments  Both |
| **Reference Sentence:** In fact, **Lao**wang wants to **hike** on steep **mountain** trails after work.  In fact, **Lao**wang wants to **hike** on steep **mountain** trails after work.  In fact, Laowang wants to hike on **steep** mountain **trails** after **work**.  In fact, **Shen**wang wants to **run** on steep **high**ways after work.  In fact, Shenwang wants to run on **steep** high**ways** after **work**. | Baseline  Prosody  Segments  Both |
| **Reference Sentence:** No, Dingdang wants to put a **brown** wooden **table** in the **living** room.  No, Dingdang wants to put a **brown** wooden **table** in the **living** room.  No, Ding**dang** wants to **put** a brown **wooden** table in the living room.  No, Dingdang wants to put a **black** wood**carving** in the **dining** room.  No, Ding**dang** wants to **put** a black **wood**carving in the dining room. | Baseline  Prosody  Segments  Both |
| **Reference Sentence:** No, she is a new **blonde** **dramatic** actress from the **west.**  No, she is a **blonde** **dramatic** actress from the **west.**  No, **she** is a **new** blonde dramatic **actress** from the west.  No, she is a new **stage** actress with **brown** hair from the **east**.  No, **she** is a **new** stage **actress** with brown hair from the east. | Baseline  Prosody  Segments  Both |
